# Tailored Strategies and Shared Decision-Making for Caregivers Managing Patients’ Pain: A Web App

**DOI:** 10.1093/geroni/igaa057.498

**Published:** 2020-12-16

**Authors:** Nai-Ching Chi, Lynn Nakad, Ying-Kai Fu, Ibrahim Demir, Stephanie Gilbertson-White, Keela Herr, George Demiris, Lee Burnside

**Affiliations:** 1 University of Iowa, Iowa City, Iowa, United States; 2 University of Pennsylvania, Philadelphia, Pennsylvania, United States; 3 University of Washington, Seattle, Washington, United States

## Abstract

Family caregivers (FCs) are essential in helping manage pain for patients who receive hospice care at home, but an accessible resource to prepare FCs in pain management is lacking. This study aims to develop an accessible resource that aids FCs in pain management. First, we conducted two literature reviews and two secondary data analyses to investigate the challenges that FCs face when managing pain for patients. Based on the results, we identified 20 common challenges that prevent FCs from providing effective pain management. Second, we developed evidence-based content to address each identified challenge, and a library to provide FCs with pain education. Third, experts and clinicians (n=10) in hospice care validated the content, and FCs (n=10) gave feedback on how to improve the understandability of the content. Currently, we are converting the content to a web app prototype. The web app will consist of: (1) an assessment tool with questions to assess the FCs’ challenges when managing patients’ pain, (2) computer-generated strategies tailored to address the identified challenges, (3) discussion questions for FCs to use with their care team to prioritize goals of pain management, and (4) a library to enhance access to educational materials. This study presented the process of developing an evidence-based and user-centered web app. This study will advance the field of pain management in hospice care by providing accessible and evidence-based tools to support and engage FCs in pain management. Also, this web app will allow best practices to be quickly translated from research into practice.

